# Short Telomeres in Hatchling Snakes: Erythrocyte Telomere Dynamics and Longevity in Tropical Pythons

**DOI:** 10.1371/journal.pone.0007493

**Published:** 2009-10-16

**Authors:** Beata Ujvari, Thomas Madsen

**Affiliations:** 1 School of Biological Sciences, University of Wollongong, Wollongong, New South Wales, Australia; 2 Evolutionary Biology Unit, Australian Museum, Sydney, New South Wales, Australia; 3 Department of Animal Ecology, Lund University, Lund, Sweden; 4 Animal Ecology Research Group, Hungarian Academy of Science, Hungarian Natural History Museum, Budapest, Hungary; Smithsonian Institution National Zoological Park, United States of America

## Abstract

**Background:**

Telomere length (TL) has been found to be associated with life span in birds and humans. However, other studies have demonstrated that TL does not affect survival among old humans. Furthermore, replicative senescence has been shown to be induced by changes in the protected status of the telomeres rather than the loss of TL. In the present study we explore whether age- and sex-specific telomere dynamics affect life span in a long-lived snake, the water python (*Liasis fuscus*).

**Methodology/Principal Findings:**

Erythrocyte TL was measured using the Telo TAGGG TL Assay Kit (Roche). In contrast to other vertebrates, TL of hatchling pythons was significantly shorter than that of older snakes. However, during their first year of life hatchling TL increased substantially. While TL of older snakes decreased with age, we did not observe any correlation between TL and age in cross-sectional sampling. In older snakes, female TL was longer than that of males. When using recapture as a proxy for survival, our results do not support that longer telomeres resulted in an increased water python survival/longevity.

**Conclusions/Significance:**

In fish high telomerase activity has been observed in somatic cells exhibiting high proliferation rates. Hatchling pythons show similar high somatic cell proliferation rates. Thus, the increase in TL of this group may have been caused by increased telomerase activity. In older humans female TL is longer than that of males. This has been suggested to be caused by high estrogen levels that stimulate increased telomerase activity. Thus, high estrogen levels may also have caused the longer telomeres in female pythons. The lack of correlation between TL and age among old snakes and the fact that longer telomeres did not appear to affect python survival do not support that erythrocyte telomere dynamics has a major impact on water python longevity.

## Introduction

One of the fundamental factors determining longevity is the ageing process. Because of its obvious negative impact on organismal fitness, it should be opposed by natural selection. Recent research demonstrate that ageing, and longevity are also governed by genetical processes [1] which have been shown to consist of several factors, such as mitochondrial mutations, modulations of stem cell developmental pathways and immunosenescence [2], [3]. Another fundamental cellular process that has been linked to aging is the gradual attrition of telomeres with increasing age [4].

Telomeres are the physical ends of linear chromosomes. The primary function of these DNA motifs, and their associated proteins, is to prevent the end of the chromosomes from being treated as DNA double-strand breaks, which would be subject to fusions and rearrangements ultimately resulting in cell-cycle arrest [5]. In vertebrates, telomeres are composed of variable numbers of tandem repeats (TTAGGG nucleotides), which have been highly conserved during vertebrate evolution [6]. In human somatic cells, telomeres become shorter with each replication [7], ultimately resulting in critically shortened telomeres, and concomitant replicative senescence [8]. This unique feature of telomeres is described by the “telomere hypothesis of cellular aging” which proposes that telomeres serve as a “mitotic clock” [5].

In humans, telomeres are known to shorten with increasing age [9], and similar results have been obtained in studies of telomere length (henceforth TL) of some birds and in garter snakes [10], [11]. However, in some long-lived birds TL does not appear to decrease with increasing age [12] and therefore in the present study, using both cross-sectional and longitudinal data, we explore age- and sex-specific telomere dynamics in another long-lived organism, the water python.

## Results

### Cross sectional analyses

**Figure 1:**
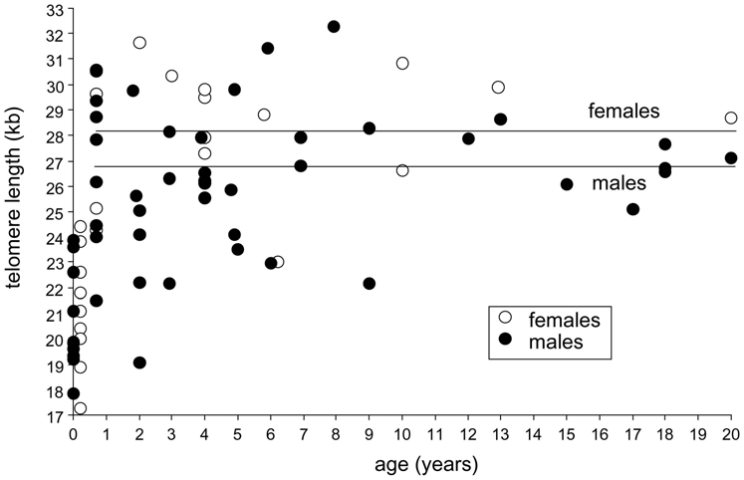
Age-specific cross-sectional comparison of male and female python telomere length. The two lines depict male and female telomere length based on an ANCOVA analysis from which hatchlings were excluded.

**Figure 2:**
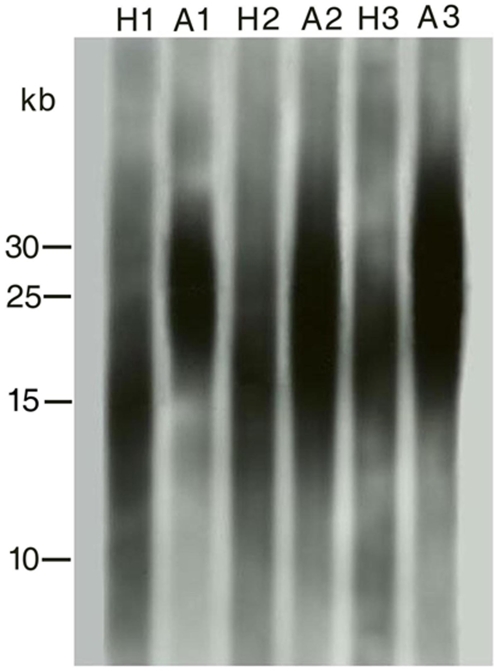
Telomere gel showing telomere length of three hatchlings (depicted H1, H2 and H3, see table 1) and their corresponding telomere length when recaptured as adults (four years old, depicted A1, A2 and A3). Location of kb markers are shown along the left-hand side of the gel.

When hatchling pythons were included in an analysis of the relationship between TL and age a significant positive correlation was revealed (Spearman rank correlation: *r_s_* = 0.57, *p*<0.0001, *n* = 70). However, when the hatchlings were excluded, the same analysis did not support any relationship between TL and python age (Spearman rank correlation: *r_s_* = 0.06, *p* = 0.68, *n* = 58, as this analysis was restricted to older snakes it included the eight recaptured pythons). Thus, based on cross sectional data TL did not change in pythons ranging in age from between 1 and 20 years of age (Fig. 1). We therefore performed a separate analysis of TL in hatchlings compared to that of one-year old snakes. An unpaired t-test revealed a highly significant difference in TL between the two groups (*t_31_* = 6.83, *p*<0.0001, Figs. 1 and 2). The mean TL of the hatchlings was approximately 6.5 kb shorter than that of the one-year old snakes (mean: 20.62 kb and 27.05 kb, respectively).

No difference in TL was observed among male and female hatchlings (unpaired t-test: *t_18_* = 0.87, *p* = 0.40, mean 20.20 kb and 21.15 kb, respectively). However, in older pythons a one-factor ANCOVA revealed a significant difference in male and female TL (*F_1,55_* = 6.26, *p*<0.015, sex as factor, age as covariate and TL as dependent variable, Fig. 1). The TL of female pythons was approximately 2 kb longer across all ages than that of the males (mean: 28.52 kb and 26.60 kb, respectively, Fig. 1).

### Longitudinal analyses

The TL of all the eight hatchlings recaptured at an older age increased substantially with increasing age (six recaptured at an age of four years and two recaptured at an age of 10 years, mean increase in TL: 7.51 kb, Table 1, Fig. 2). However, in older snakes TL decreased with increasing age in all of the 13 pythons (Table 2), but we did not detect any association between telomere attrition rate and python age (Spearman rank correlation: *r_s_* = 0.27, *p* = 0.35, *n* = 13). Furthermore, among-year telomere attrition rate differed substantially in five of the six snake recaptured on more than one occasion (Table 2).

**Table 1:** Telomere dynamics within 8 hatchling water pythons re-sampled at an age of four and 10 years.

python	age (years)	sex	TL (kb)	difference in TL (kb)
1	0	M	15.26	
1	4		26.51	+11.25
2	0	M	19.86	
2	4		25.54	+5.68
3	0	F	20.01	
3	4		29.81	+9.80
4	0	F	21.80	
4	4		27.92	+6.12
5	0	F	17.26	
5	4		29.51	+12.25
6	0	M	23.86	
6	4		26.22	+2.36
7	0	F	20.40	
7	10		26.61	+6.21
8	0	F	24.43	
8	10		30.84	+6.91

**Table 2:** Telomere dynamics within 13 water pythons initially sampled at ages from one to 18 years.

python	age (years)	sex	TL (kb)	difference in TL (bp)
1	1	F	24.28	−960
1	2		23.32	−400
1	3		22.92	
2	1	M	24.02	−10
2	2		24.01	
3	2	M	28.12	−1550
3	3		26.57	−360
3	4		26.21	
4	2	M	25.05	−40
4	3		25.01	−840
4	4		24.17	
5	2	F	29.51	−1100
5	3		28.41	−1080
5	4		27.33	
6	2	M	19.86	−780
6	3		19.08	
7	2	M	22.22	−730
7	5		21.49	
8	3	M	26.01	−830
8	4		25.18	−1660
8	5		23.52	
9	3	F	23.19	−160
9	6		23.03	
10	4	M	23.47	−360
10	5		23.11	−140
10	6		22.97	
11	9	M	28.26	−440
11	10		27.82	−810
11	11		27.01	
12	11	M	28.16	−282
12	12		27.88	
13	18	M	26.77	−60
13	19		26.71	

### TL and survival

When using recapture as a proxy for python survival, we did not observe any significant difference in mean TL of recaptured versus non-recaptured hatchling pythons *t_18_* = 0.39, *p* = 0.70; mean TL 20.36 kb and 20.80 kb, respectively). However, the TL of non-recaptured older pythons was significantly longer than those recaptured *t_48_* = 3.19, *p* = 0.0025; mean TL 27.61 kb and 24.90 kb, respectively).

## Discussion

In contrast to most other vertebrates, TL of hatchling pythons was significantly shorter than that of older snakes. Recently, similar results have been presented in a study of TL in a long-lived sea bird, the Leach's storm petrel (*Oceanodroma leucorhoa*) [13]. However, this study is only based on cross sectional data, and hence needs longitudinal data to confirm the results obtained. The difference in TL between hatchling pythons and older snakes is unlikely to be caused by selective mortality as our longitudinal data demonstrated that the TL increased substantially when the snakes were re-sampled at an older age. The dramatic difference in TL between hatchlings and one-year-old snakes suggests a rapid, and substantial increase in TL (approximately 7 kb) during the python's first year of life. In fish, high telomerase activity has been reported in all somatic tissues examined, and the reason for this up-regulation has been suggested to be due to high somatic cell proliferation rates [14]. Water pythons also exhibit an extremely rapid increase in body length during this period [15], requiring a very high cell proliferation capacity. We therefore suggest that the increase in TL during the python's first year of life may have been caused by up-regulation of telomerase activity in the hematopoietic stem cells. However, growth rate in numerous other young organisms, such as humans is also very rapid [9], but in spite of this TL in newborn humans shortens rapidly during their first years of life [9]. Thus, further studies are indeed crucial in order to elucidate mechanism(s) involved in the rapid increase in TL in hatchling pythons.

When hatchling pythons were excluded from the cross-sectional analyses, we did not observe any correlation between TL and python age. Our results thus mirror those obtained in studies of age-specific TL dynamics in long-lived birds [12]. However, in spite of the lack of cross-sectional age-dependent decline in python TL, our longitudinal analyses demonstrated that TL in older pythons did decrease with increasing age, albeit at highly variable rates. This variability is not likely to have been caused by environmental effects such as annual variation in climate and/or prey availability as during the same year we observed highly variable among-individual decrease in TL. Thus, solely relying on cross-sectional data would not have provided a correct picture of older water python's telomere dynamics.

Studies conducted on TL in newborn humans have revealed no sex-specific differences [16], whereas in older humans, female telomeres are longer compared to males [9]. The results obtained in the present study mirror those obtained in studies of humans, i. e. absence of sex-specific differences in TL among the hatchlings, and older female pythons having longer telomeres than males across all ages.

The disposable soma theory has been suggested as a possible evolutionary background for longer telomeres in females as compared to males in ants, rats and humans [17]. This theory rests on the idea that organisms subjected to high levels of extrinsic mortality should be selected to invest less in somatic maintenance, resulting in shorter intrinsic lifespan [18]. However, although female pythons have longer telomeres than males, due to higher costs of reproduction, females have higher mortality rates, and hence shorter lifespan compared to male pythons [19]. Thus, the disposable soma theory is unlikely to explain the sex-specific difference in TL in water pythons.

Two additional hypotheses have been proposed to explain the sex-specific difference in human TL. (1) X-inactivation with advancing age in heterozygous women might be preferentially skewed to implicate the allele harbouring the genetic code that results in short telomeres [20]. However, in contrast to humans, female snakes are heterogametic [21], thus sex chromosome inactivation is unlikely to have caused the relatively longer telomeres in the female pythons. (2) In humans, estrogen responsive elements present in hTERT promotor may stimulate increased telomerase activity which has been suggested to result the longer telomeres in females [22], and we therefore suggest that the effect of estrogen on telomerase activity may account for the relatively longer telomeres in female pythons.

In birds erythrocyte TL has been found to be associated with fitness and life span [23], [24], and similar results have been obtained when studying the effect of leukocyte TL on human longevity [25]. However, no association between leukocyte TL and age was detected in a study of old humans [26]. Furthermore, replicative senecence has been shown to be induced by a change in the protected status of the shortened telomeres rather than by the loss of telomeric DNA [27]. Four of our results do suggest that telomere dynamics did not have a significant impact on python longevity: (1) The lack of correlation between TL and age among the older pythons, (2) the large among year variation in telomere attrition rate of snake recaptured over several years, (3) the lack of a difference in TL among the recaptured versus non-recaptured hatchling pythons, and (4) significantly longer telomeres were observed in the non-recaptured than in the recaptured older water pythons. Thus, at present, the issue of how and under what circumstances telomere dynamics, and replicative senescence affect organismal longevity remains unsettled.

## Materials and Methods

### Study species

Water pythons are large (up to 3 m), non-venomous snakes widely distributed across tropical Australia [28]. Our analyses are based on 70 individually marked pythons for which we could confidently infer the age of each snake. These animals fall into two groups: (1) offspring from eggs hatched in our laboratory, and (2) hatchlings and juveniles (<1 year old) collected in the field. During their first year the small size of the juvenile pythons (<110 cm) results in that age can confidently be determined [15]. Twenty of 70 pythons were hatchlings (11 males and 9 females) randomly sampled from 20 different broods, and hatched in our laboratory in 1992 and in 2003. Eight of the 20 hatchlings (five females and three males) were recaptured at an age of four and 10 years. The 50 older snakes (39 males and 11 females) were captured in between 2004 and 2007 at our study site, the Fogg Dam Conservation Reserve, situated 60 km south east of Darwin in the Northern Territory of Australia.

### DNA extractions and TL measurements

Blood samples (100 µl) were collected by cutting off the last 2 mm of the python's tail. All blood samples were stored in 70% ethanol at −20°C during the field season (approximately four months). The DNA was then extracted within a week or two after samples were brought back to our laboratory, and stored in TE buffer at −80°C until used. Nucleated erythrocytes were separated by centrifugation at 6000 rpm. Erythrocyte genomic DNA was extracted by phenol-chloroform extraction [29]. Telomere restriction fragment (TRF) length was measured by Southern blot hybridisation following the protocol outlined in the Telo TAGGG TL Assay Kit (Roche). Constant field electrophoresis was used, and the gels were run for 24 hours at 50 volts. We used two molecular weight marker (1) the one provided by the TeloTAGGG Telomere Length Assay kit (Roche), and (2) a high molecular weight DNA Markers, (Cat No: 15618-010, Invitrogen; size range: 8.2– 48.5 kb). Using a digoxigen-labled probe, the image of the TRF smears were subsequently developed on high performance chemiluminescence film (Amersham Bioscience). As erythrocytes are descendants from hematopoietic stem cells of different replicative history, resulting in variation of TL in each sample, the intensity of the entire TRF smears representing the genome-wide TL was measured on a GS-800 densitometer using Quantity One software (BioRad). Background signal was defined as the level of “noise” at the higher molecular weight smear i.e. “above” of the telomere “peak” quantified by the GS-800 densitometer, and subsequently subtracted from the signal intensity of each lane prior to TL calculation. Mean TRF length of telomere distributions was calculated using the formula: *L* = Σ(OD_1_ *L*_1_)/Σ(OD_1_), where OD_1_ is the signal intensity at position 1 and *L*_1_ is the length of the DNA (bp) at position 1. On each gel one of the pythons was run as control for differences between assays. Furthermore, each sample was run on two separate gels. TL of the two samples were highly correlated (*r* = 0.98, *p*<0.001, *n* = 70), and the average values from runs per sample were used in subsequent analyses.

The work was conducted under University of Wollongong animal ethics approval number AE04/03, and according to the Parks and Wildlife Commission of the Northern Territory permit number 21940.

### Statistical analyses

Parametric statistics were used in the analyses when our data confirmed to normality (Shapiro and Wilke's test, *P*>0.05), whereas non-parametric statistics were employed when these requirements were not fulfilled.
